# Matriptase Deletion Initiates a Sjögren’s Syndrome-Like Disease in Mice

**DOI:** 10.1371/journal.pone.0082852

**Published:** 2014-02-13

**Authors:** Hongen Yin, Peter Kosa, Xibao Liu, William D. Swaim, Zhennan Lai, Javier Cabrera-Perez, Giovanni Di Pasquale, Indu S. Ambudkar, Thomas H. Bugge, John A. Chiorini

**Affiliations:** 1 Molecular Physiology and Therapeutics Branch, National Institute of Dental and Craniofacial Research, National Institutes of Health, Bethesda, Maryland, United States of America; 2 Oral and Pharyngeal Cancer Branch, National Institute of Dental and Craniofacial Research, National Institutes of Health, Bethesda, Maryland, United States of America; Biological Research Centre of the Hungarian Academy of Sciences, Hungary

## Abstract

**Objective:**

The objective of this study was to determine the effect of epithelial barrier disruption, caused by deficiency of the membrane-anchored serine protease, matriptase, on salivary gland function and the induction of autoimmunity in an animal model.

**Methods:**

Embryonic and acute ablation of matriptase expression in the salivary glands of mice was induced, leading to decreased epithelial barrier function. Mice were characterized for secretory epithelial function and the induction of autoimmunity including salivary and lacrimal gland dysfunction, lymphocytic infiltration, serum anti-Ro/SSA, anti-La/SSB and antinuclear antibodies. Salivary glands immune activation/regulation, barrier function as well as tight junction proteins expression also were determined. Expression of matriptase in minor salivary gland biopsies was compared among pSS patients and healthy volunteers.

**Results:**

Embryonic ablation of matriptase expression in mice resulted in the loss of secretory epithelial cell function and the induction of autoimmunity similar to that observed in primary Sjögren’s syndrome. Phenotypic changes included exocrine gland dysfunction, lymphocytic infiltrates, production of Sjögren’s syndrome-specific autoantibodies, and overall activation of the immune system. Acute ablation of matriptase expression resulted in significant salivary gland dysfunction in the absence of overt immune activation. Analysis of the salivary glands indicates a loss of electrical potential across the epithelial layer as well as altered distribution of a tight junction protein. Moreover, a significant decrease in matriptase gene expression was detected in the minor salivary glands of pSS patients compared with healthy volunteers.

**Conclusions:**

Our findings demonstrate that local impairment of epithelial barrier function can lead to loss of exocrine gland dysfunction in the absence of inflammation while systemic deletion can induce a primary Sjögren’s syndrome like phenotype with autoimmunity and loss of gland function.

## Introduction

Primary Sjögren’s syndrome (pSS) is a chronic autoimmune disease that primarily affects lacrimal and salivary glands, leading to dry eyes and mouth, but it can also affect lungs, kidneys, skin, and thyroid. Like most rheumatic diseases, pSS lacks a single distinguishing feature or diagnostic test but relies on a combination of clinical and laboratory findings including ocular and oral symptoms, lymphocytic infiltration of salivary glands, and a presence of autoantibodies [1].

Tight junctions are the primary structures that separate the apical and basolateral membrane regions of the cell, and together with several other molecules, such as claudins, occludins, and zona occludens, are central to the barrier function of an epithelial monolayer [2], [3]. Several studies in humans and animals with primary epithelial barrier defects have underscored the importance of the integrity of these tight junctions as regulators of the exposure of microbiota to resident immune cells and immune tolerance [4], [5]. Indeed, genetic defects in the epithelial barrier cells are linked to a spectrum of allergic and autoimmune diseases [6]–[17]. In this respect, the etiology of pSS is still incompletely understood and both epithelial dysfunction and primary immune defects have been proposed as initiating factors.

We recently reported that mice with salivary gland deletion of the protease matriptase, which is important for maintaining epithelial barrier function, display a significant decrease in pilocarpine-stimulated saliva production [18]. This observation suggests a critical role of matriptase in the maintenance of the salivary gland function. In the present study, we therefore have further investigated the possible contribution of compromised epithelial barrier function to the initiation of pSS, and the relationship between matriptase expression, epithelial barrier function of exocrine glands, and pSS-like disease development. Mice with reduced matriptase expression displayed a number of characteristics associated with pSS in human patients, including impaired salivary and lacrimal glands function, lymphocytic infiltrates, global immune activation, and elevated autoantibodies associated with pSS. Our data also showed altered distribution of cell junction protein claudin 3 along with impaired epithelial barrier function. Moreover, gene expression analysis of human minor salivary glands demonstrated significantly decreased matriptase levels in pSS patients compared with healthy volunteers. Our study provides the first demonstration of a link between altered expression of an epithelial barrier-associated protein and salivary gland function.

## Materials and Methods

### 1. Ethics statement

The minor salivary gland samples were obtained from the NIDCR Sjögren’s syndrome clinic. The study was approved by the Institutional Review Board of the National Institute of Dental and Craniofacial Research protocol numbers: 94-D-0018, 84-D-0056 and 99-D-0070. Informed consent was obtained in writing from all study subjects prior to enrollment.

All mouse studies were conducted in an AAALAC accredited facility under the NIH, NIDCR Institutional Animal Care and Use Committee Protocol approval. The use of mice was selected because no non-mammalian test or in vitro method to the current knowledge can provide the same degree of specificity and reliability as the mammalian model.

### 2. Animals

All studies were strictly littermate controlled. Matriptase conditional knockout mice, hereafter termed “*St14^LoxP/LoxP^*” and matriptase knockout mice (*MMTV-Cre* ^+/0^;*St14^LoxP/−^*, hereafter termed “*St14^−^*” mice), along with respective controls (*MMTV-Cre* ^+/0^;*St14^LoxP/+^*, *MMTV-Cre* ^0^;*St14^LoxP/−^*, and *MMTV-Cre* ^0^;*St14^LoxP/+^*, hereafter termed “*St14^+^*” mice) were described previously [18]. All experimental animals were in a mixed 129/C57BL6/J/NIH Black Swiss/FVB/NJ background. The genotypes of all mice were determined by PCR of ear or tail biopsy DNA as described previously [18]. Pilocarpine stimulated salivary and tear flow rate were measured as described [19], [20].

### 3. Cell Lines

HEK-293T cells were grown in Dulbecco’s modified Eagle’s medium supplemented with 10% heat-inactivated fetal bovine serum (Life Technologies, Rockville, MD, USA), 2 mM L-glutamine, penicillin (100 U/ml), and streptomycin (100 µg/ml) (Biofluids, Rockville, MD, USA) as previously described [21].

### 4. AAV2 vector administration

To generate salivary gland-specific deletion of matriptase in adult animals, adeno-associated virus (AAV2) vector encoding the 1.8×10^11^ particles/mouse (0.9×10^10^ particles/gland) Cre recombinase or beta galactosidase was delivered into the submandibular salivary glands of *St14^LoxP/LoxP^* mice by retrograde instillation as previously described [22] hereafter termed AAV2-Cre or AAV2-LacZ mice, respectively. A lower dose of AAV2-luciferase (5×10^8^particles/gland) was co-administered to enable monitoring of the expression of the AAV2 vectors by live imaging of the animals (Figure S1A).

### 5. Measurement of salivary and tear flow rate and regulated volume decrease

To determine the salivary and lacrimal gland secretion, pilocarpine stimulated salivary flow rate (SFR) and tear flow rate (TFR) were measured as described [19], [20].

Regulated volume decrease (RVD) of ductal and acinar cells from submanibular salivary glands (SMG) of AAV2-Cre and AAV2-LacZ treated mice was measured as described [23]. RVD was induced by addition of hypotonic solution of 150 mOsm and relative cell volume was measured before and after hypotonic solution stimulation.

### 6. Lymphocytic infiltration in the exocrine glands and serum autoantibodies detection

24–55 wks old *St14^−^* and *St14^+^* mice were euthanized and lymphocytic foci (LF) were detected by H&E staining of salivary and lacrimal glands. The total SMG area was measured using a calibrated ocular with 4 mm^2^ square sections as previously described [24]. Slides were scanned by the Aperio ImageScope (Vista, CA) and the area of the lymphocytic infiltration was counted by Positive Pixel Count Algorithm (9.1, Aperio ImageScope, Vista, CA).

Serum was collected from 24–54 week old *St14^+^* and *St14^−^* animals. The detection of anti-SSA/Ro [multiple antigenic peptide (MAP)-Ro273], anti-SSB/La and anti-nuclear antibody (ANA) were measured as described previously [19], [24].

### 7. Flow cytometry analysis

T cell activation was assessed by staining the cells for CD4, CD8, and CD62L markers. One million salivary gland draining lymph node (DLN) cells or splenocytes were stained with PerCP-conjugated anti-mouse CD4 or APC-conjugated anti-mouse CD8 with PE-conjugated anti-mouse CD62L (BD Pharmingen, San Diego, CA, USA). Data were acquired using a FACSCalibur instrument (BD Biosciences, San Diego, CA, USA), and analyzed using Flowjo software (Tree Star Inc., Ashland, OR, USA). Flow cytometric identification of CD4+CD25+ Foxp3+ T regulatory (Treg) cells was performed as described previously [25].

### 8. Cytokine and chemokine detection

Cytokines released from cultured DLN cells and splenocytes and serum cytokines were detected as described previously and measured using a multiplex sandwich-ELISA assay (Aushon Biosystem Billerica, MA, USA) [20]. Briefly, splenocytes and salivary gland-associated DLN cells obtained from AAV2-treated mice were isolated and samples from two mice in each group were pooled and cultured in 24-well plates at 5×10^6^ cells/mL RPMI-1640 medium (Invitrogen, Carlsbad, CA), containing HL-1 serum replacement (Cambrex Bioscience, Walkersville, MD), with or without 1 µg/mL Concanavalin A (ConA, Sigma-Aldrich, St. Louis, MO).

### 9. Transepithelial electrical potential (EP) measurement

To measure the EP, a high impedance electrode attached to a dual differential Electrometer FD223 (World Precision Instruments, Sarasota, FL, USA) was placed in a 1 mL syringe connected to a fine cannula filled with isotonic saline (Aqualite System, Hospira, Lake Forest, IL, USA). The other end of the cannula was inserted in the submandibular duct by retrograde cannulation. The EP was measured by placing the ground electrode on the tissue adjacent to the duct opening.

### 10. Immunofluorescent measurements

5-µm sections of paraffin-embedded SMG were processed as previously described (31). Primary antibodies were used at: 5 µg/ml rabbit anti-claudin2 and 3 (Zymed/Invitrogen) 5 µg/ml rabbit anti-MaxiK (Alomone), 10 µg/ml rabbit anti-occludin (Zymed/Invitrogen), 5 µg/ml rabbit anti-ZO-1 (Zymed/Invitrogen), 1∶500 rabbit anti-TMEM16A (a gift from Dr. James Melvin, NIDCR, NIH), and 1∶200 dilution of rabbit anti-NKCC1 (a gift from Dr. James Turner, NIDCR, NIH) all in 0.5% BSA/PBS followed by a 1∶100 dilution of donkey anti-rabbit IgG Alexa 488 (Invitrogen) in 0.5% BSA/PBS.

### 11. Minor salivary gland samples from patients and healthy volunteers

Minor salivary gland biopsies from the lower lip of female pSS patients (21–60 years old) along with female healthy volunteers (22–69 years old) were obtained in accordance with the European-American consensus group criteria [26].

### 12. Microarray analysis

Microarray analysis of matriptase expression was derived from our study of the global gene expression changes in minor salivary glands from pSS patients (data unpublished) using custom 4×44K microarray slides (Agilent Technologies, Santa Clara, CA). Microarrays were hybridized according to the manufacturer’s recommendations for One-Color Microarray-Based Gene Expression Analysis and scanned using an Agilent G2565AA microarray scanner (Agilent Technologies) and analyzed using GeneSpring software (Agilent Technologies) or Ingenuity Pathway Analysis. Microarray data was deposited with the Gene Expression Omnibus (GEO) database the accession number is GSE51016.

### 13. Quantitative PCR analysis

Quantitative PCR (qPCR) analysis of matriptase expression was performed as described previously [18]. Each PCR reaction was run in triplicate. Matriptase expression levels were normalized to glyceraldehyde-3-phosphate dehydrogenase (*GAPDH*) levels in each sample. Human *ST14* gene expression is represented by the fold change, which was calculated as 2*^−^* ^ΔΔCT^, while mouse *St14* expression was presented as ΔΔCT, which was described in the manufacturer’s instruction (http://www3.appliedbiosystems.com/cms/groups/mcb_support/documents/generaldocuments/cms_042380, Page 58–59).

### 14. Statistical analysis

Statistical significance between experimental groups was assessed using unpaired Student’s t-test and Mann-Whitney u test for comparison of LG lymphocytic infiltration area between the *St14^+^* and *St14^−^* mice. Correlation of SFR and EP was analyzed by Pearson’s rank correlation test (GraphPad Software Inc., La Jolla, CA, USA). A P value ≤0.05 was considered statistically significant.

## Results

### 1. Loss of exocrine gland function correlates with the loss of matriptase expression in mice

We have previously shown that embryonic deletion of salivary gland matriptase in mice leads to a near-complete loss of saliva production [18]. To further investigate this observation and the possible link between compromised salivary gland epithelial barrier, we performed a detailed analysis of two cohorts: (i) cohort A consisted of *St14^−^* mice with mouse mammary tumor virus (MMTV)-Cre-driven embryonic deletion of matriptase and control *St14^+^* mice; (ii) cohort B consisted of *St14^LoxP/LoxP^* mice with AAV2-mediated expression of either Cre recombinase (AAV2-Cre mice) or β-galactosidase (AAV2-LacZ mice, control). *St14^−^* mice display embryonic loss of matriptase, while treatment of the submandibular salivary glands of *St14^LoxP/LoxP^* mice with AAV2-Cre vector leads to acute deletion of matriptase in adult mice. The route of delivery and tropism of AAV2 vector results in transduction of ductal cells and confined localization of the vector in the salivary glands [27] (Figure S1A). Live imaging showed high levels of luciferase expression (all tested as >10^7^/mouse) indicating delivery of the AAV2 vectors to both submandibular salivary glands (Figure S1A). Using qPCR analysis, we showed that matriptase mRNA level in AAV2-Cre mice was reduced by 82.5% compared with control AAV2-LacZ animals (Figure S1B). This result is comparable with our previous observation of an 80% reduction in matriptase mRNA expression in mice with embryonic deletion of matriptase in the salivary glands [18].

**Figure 1:**
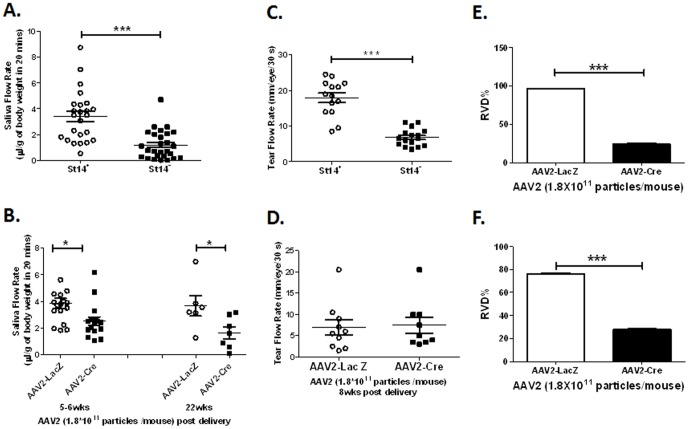
Exocrine gland dysfunction in matriptase-deficient mice. Pilocarpine-stimulated salivary flow rate (SFR) and tear flow rate (TFR) were measured in mice with either embryonic or acute salivary gland ablation of matriptase. (A, C) At 24–54 weeks of age, *St14^–^* mice showed a significant reduction in SFR as well as TFR (*N* = 27 for SFR and *N = *16 for TFR) compared with the littermate *St14^+^* control mice (*N* = 24 for SFR and *N* = 14 for TFR; ***P<0.0001). (B, D) AAV2-mediated acute deletion of matriptase led to a significant decrease in SFR in AAV2-Cre animals (*N = *17) compared with the AAV2-LacZ controls (*N = *17) 5–6 weeks after AAV2 administration (*P = 0.0126). This decrease in saliva production was stable for at least 22 weeks post AAV2 delivery (*N = *6 in AAV-LacZ and *N = *7 in AAV-Cre group; *P = 0.037). At 8 weeks post-AAV2 delivery, TFR was measured revealing no significant changes between matriptase-deficient and control animals (*N = *10 for AAV2-LacZ and *N = *10 for AAV2-Cre, *P* = 0.8353). (E, F) SMG ductal and acinar cells RVD% was calculated as the relative cell volume changes after hypotonic solution stimulation. Data shown are the mean ± SEM for each group. Statistical significance was determined using an unpaired Student’s two-tailed *t* test. ***P<0.0001.

We next assessed SFR and TFR in both cohorts after injection of pilocarpine. The SFR (mean ± SEM) of 24–54-week old *St14^−^* animals compared with littermate *St14^+^* controls was significantly reduced (1.2±0.2 µl/g 20 min versus 3.40±0.4 µl/g body weight in 20 min, respectively; *P*<0.0001) (Figure 1A). Furthermore, the analysis of TFR in this cohort showed a significant decrease in tear production in matriptase-deficient *St14^−^* animals compared with the *St14^+^* control group (6.8±0.6 mm/30 s and 18.0±1.3 mm/30 s, respectively; *P*<0.0001) (Figure 1C). Acute deletion of matriptase in adult AAV2-Cre animals resulted in similar reduction of saliva production. The SFR assessed 5–6 weeks after the vector administration was significantly lowered in AAV2-Cre mice compared with the AAV2-LacZ control mice (2.5±0.3 µl/g 20 min and 3.9±0.4 µl/g 20 min, respectively; *P* = 0.0126) and this phenotype was stable for at least 22 weeks after the vector delivery (Figure 1B). In contrast to our observation in cohort A, AAV2-Cre animals did not show any significant reduction in tear production compared with control AAV2-LacZ animals at 8 weeks (Figure 1D), further confirming the restricted localization of AAV2 vector in the salivary glands and the importance of matriptase in salivary gland function.

Regulation of fluid movement and cell volume critically impacts salivary gland fluid secretion following stimulation of the gland [28]. Incubation of both acinar and ductal cells in hypotonic solution led to an initial increase in cell volume, followed by activation of RVD and a subsequent cell volume decrease [23]. Comparison of cells isolated from the salivary glands showed a significant inhibition of volume recovery following hypotonic solution-induced osmotic stress in ductal cells from AAV2-Cre mice compared with the AAV2-LacZ control mice (mean ± SEM RVD%: 24.0±0.6 and 96.2±0.3%, respectively; *P*<0.0001) (Figure 1E). Although AAV2 expression is restricted to ductal cells (38), a significant decrease was also observed in acinar cells (27.7±0.6% and 75.8±0.9%, respectively; *P*<0.0001) (Figure 1F).

### 2. Lymphocytic infiltrates in exocrine glands and serum autoantibody production in *St14^−^* animals

**Figure 2:**
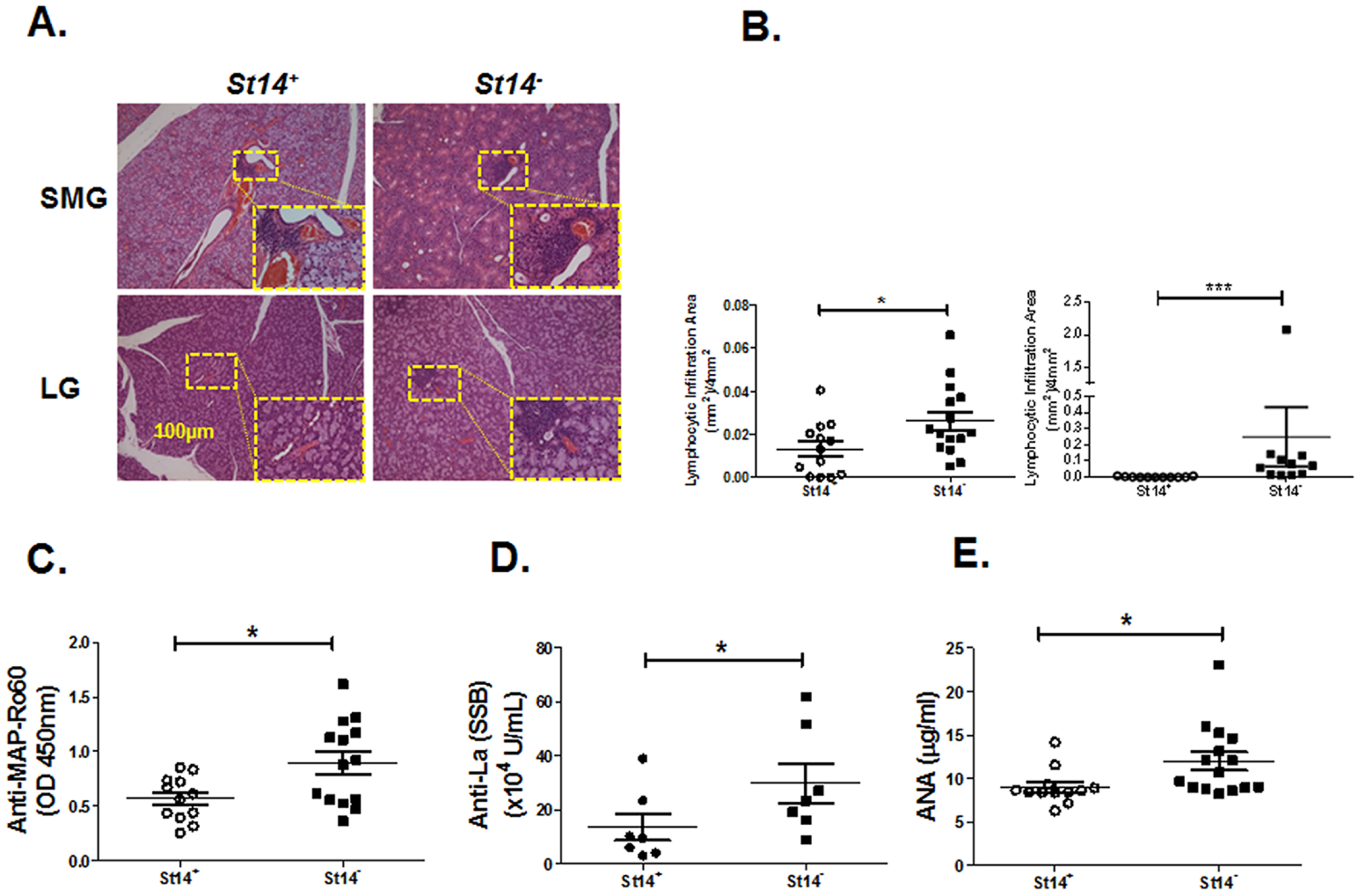
Lymphocytic infiltration and autoantibody production in the exocrine glands *St14* *^−^* mice. A) Representative lymphocytic foci (LF) were detected by H&E staining of salivary and lacrimal glands dissected from *St14^–^* and *St14^+^* mice (*N = *15 and 13 respectively for SMG and *N = *11 in both groups for LG). B) A statistically significant increase in lymphocytic infiltration area in the SMG (*P* = 0.0280) and in the lacrimal glands from *St14^–^* mice compared with *St14^+^* controls (***, *P*<0.0001). The lymphocytic infiltration area was calculated per 4 mm^2^. Serum samples were collected from both *St14^+^* (*N* = 11–12) and *St14^–^* (*N = *12–15) animals and analyzed for the presence of autoantibodies by ELISA. Significant increases in (C) anti-SSA/Ro (SSA), (D) anti-SSB/La and (E) anti-nuclear (ANA) antibody were detected in *St14^–^* mice compared with *St14^+^* mice (*, *P* = 0.0145, *, *P* = 0.045 and *, *P* = 0.0313, respectively). Data shown are the mean ± SEM for each group. Statistical significance was determined using Mann-Whitney u test for lymphocytic infiltration area in LG comparison and an unpaired Student’s two-tailed *t* test for the rest tests.

We performed a histomorphometric analysis of lymphocytic foci in lacrimal and salivary glands of *St14^−^* and *St14^+^* mice. A significant increase in the lymphocytic infiltration area (mean ± SEM) was found in SMG and LG from matriptase-deficient *St14^−^* animals and the *St14^+^* controls (0.0262±0.0043 *vs* 0.0132±0.0034 per 4 mm^2^ for SMG and 0.2498±0.1840 *vs* 0.0025±0.0001 for LG, respectively) (Figure 2A, B). Moreover, our results indicated a significant (mean ± SEM) increase in serum levels of anti-SSA/Ro, anti-SSB/La, and ANA autobodies in *St14^−^* mice (0.9±0.1 [OD], 497697±73632 U/ml, and 12.0±1.0 µg/ml, respectively) compared with *St14^+^* control mice (0.6±0.1[OD], 136658±49333 U/ml, and 9.0±0.6 µg/ml, respectively; all *P*<0.05) (Figure 2C–E). In contrast, no significant changes in lymphocytic infiltration were detected in either the salivary or lacrimal glands of AAV2-Cre mice compared to the control AAV2-LacZ mice (data not shown).

### 3. Loss of CD62L and alteration of T regulatory cells in both local and systemic immune systems in *St14^−^* mice

**Figure 3:**
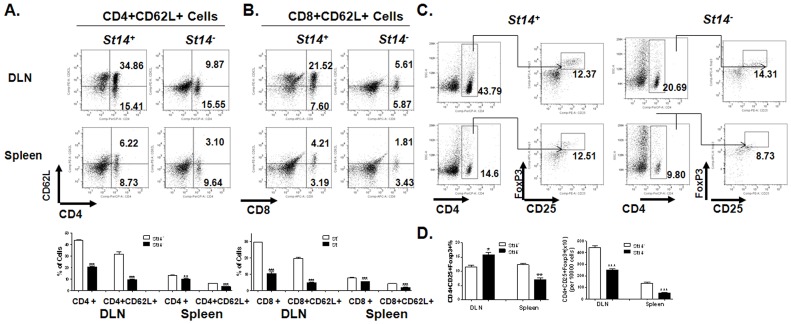
T cell activation and regulation changes in draining lymph nodes and spleens from *St14* ^–^ mice. Salivary gland draining lymph nodes (DLN) and spleens were collected from *St14^+^* and *St14^–^* animals. Samples from 2–3 mice were pooled per group and used to detect CD4, CD8 and CD62L expression by flow cytometry. (A, B) Both CD4+ and CD8+ cells were significantly decreased in both the DLN and spleens from *St14^–^* mice compared with *St14^+^* mice. CD62L expression was also decreased on both CD4+ and CD8+ cells in *St14^–^* mice compared with *St14^+^* mice. (C, D, E) The percentage and total number of CD4+CD25+Foxp3+ natural T regulatory cells (nTreg) were altered in the DLN and spleen cells. Data shown are the mean ± SEM for each animal group (*N* = 4 for both groups). Statistical significance was determined using an unpaired Student’s *t*-test. *P<0.05, **P≤0.001, ***P<0.0001.

To assay for a local immune response in salivary gland DLN cells [20], [29], as well as systemic immune response in splenocytes DLN cells and splenocytes were collected and assayed for activation. The total number of DLN cells and splenocytes was not significantly different between *St14^−^* and *St14^+^* mice (average 7×10^7^ and 9×10^7^, respectively, for DLN, and 1.5×10^8^ and 1.4×10^8^, respectively, for splenocytes) however, a remarkable decrease in the number and percentage of CD4+ and CD8+ cells was detected among DLN cells and splenocytes from *St14^−^* mice compared with controls (*St14^+^* vs *St14^−^* mice: CD4∶43.9±0.6% vs 20.6±0.4% in DLN and 13.1±0.7% vs 10.2±0.2% in spleen; CD8∶29.4±0.3% vs 10.2±0.2% in DLN and 7.8±0.1% vs 5.4±0.1% in spleen, respectively; *P*<0.0001) (Figure 3A–B). Moreover, we also detected a significant loss of CD62L (L-selectin) expression on CD4+ and CD8+ T cells from both DLN and spleenocytes from *St14^−^* mice (*St14^+^* vs *St14^−^* mice: CD4 CD62L: 31.8±1.7% vs 9.6±0.2% in DLN and 6.4±0.1% vs 3.4±0.2% in spleen; CD8 CD62L: 19.6±0.8% vs 4.6±0.5% in DLN and 4.3±0.1% vs 1.9±0.1% in spleen, respectively; *P*<0.0001) (Figure 3A–B). L-selectin is mainly expressed in the naive and central memory T-lymphocytes and acts as a “homing receptor” for lymphocytes to enter and localize in secondary lymphoid tissues to encounter antigen. Loss of CD62L is seen while these cells are activated as effector T cells [30]. Our results suggest that the majority of the T cells transited to effector T cells in DLN and spleen.

Treg cells play an important role in maintaining the immune tolerance and protection against a number of autoimmune diseases [31]. We observed a significant decrease in the CD4+CD25+ Foxp3+ Treg cell population in spleens from *St14^−^* mice compared with *St14^+^* controls (Figure 3D). In the DLN, the total number of Treg cells was also decreased in *St14^−^* mice (444.8±17.4/10^6^ tested cells vs 249.3±12.6/10^6^ tested cells, respectively; *P*<0.0001) (Figure 3D) due to the decreased number of CD4+ T cells (Figure 3B), however, the percentage of CD25+Foxp3+ cells within the CD4+ T cell population was increased compared with *St14^+^* controls (11.5±0.7% vs 15.8±1.0% in DLN from *St14^+^* and *St14^−^* mice, respectively, Figure 3D). In the systemic immune system we found a loss of Treg in the *St14^+^* mice (12.3±0.4% vs 7.1±0.5% and 135.8±9.3/10^6^ tested cells vs 53.8±3.6/10^6^ tested cells, in spleen from *St14^+^* and *St14^−^* mice, respectively).

Only minimal and statistically not significant alterations of CD4, CD8, CD62L expression and Treg numbers were observed in either DLN or spleen of AAV2-Cre mice compared to the control AAV2-LacZ mice (data not shown).

### 4. Cytokine production is increased in local and systemic immune system in *St14^−^* mice

To further characterize the immune system in the salivary glands of matriptase-deficient mice, we measured the concentration of cytokines released from cells isolated from pooled samples of salivary gland DLN from *St14^−^* and *St14^+^* mice, which is the most relevant secondary lymphoid organ tracking local inflammation [29]. We observed a significant increase in IL-1β, IL-4, IL-5, IL-6, IL-10, IL-12p40, IL-13, IL-17, and IFN-γ in the media from cell cultures isolated from *St14^−^* mice compared with *St14^+^* mice (Table 1). A slight increase in the release of MCP-1, MCP-5, MIP-1β, RANTES, and MMP-9, as well as other T cell cytokines (IL-12p70, IL-18, IL-23 and TGF-β1) was also observed, however, these differences did not reach statistical significance (Table 1).

**Table 1:** Cytokine productions in SG local and systemic immune system from St^+^ and St^-^ mice (pg/ml).

		DLN culture				Splenocyte culture				Serum				
		St^+^	St^−^	t test	change	St^+^	St^−^	t test	change	St^+^	St^−^	t test	change	LDL
**Mφ**	**IL-1β**	3.30±4.66	20.73±3.84	0.0276	↑,*	14.53±2.46	13.93±29.51	0.4900	n/a	n/a	n/a			3.10
														
**Chemokines**	**MCP-1**	3.25±21.53	1201.67±797.38	0.0838	↑	1451.92±33.19	18873.44±4980.59	0.0193	↑,*	37.67±26.84	38.5931.47	0.4855	NS	
	**MCP-5**	0.020±0.3534	9.5±0.8971	0.0026	↑	12.69±2.28	1099.57±53.72	0.0006	↑,***	30.56±8.77	54.30±36.87	0.1694	↑	
	**MIP-1β**	104.29±21.09	255.46	0.0053	↑	1926.96±308.62	7428.28±1196.84	0.0122	↑,*	6.52±0.53	5.80±1.14	0.1873	NS	
	**MMP-9**	242.70	8369.98±1271.21	0.0060	↑	0.00	18294.25±4487.75	0.0143	↑,*	6716.01±5594.15	16899.70±8880.83	0.0841	↑	20.00
	**RANTES**	174.47±39.09	211.86±32.64	0.2041	↑	1386.48±84.04	2143.47±16.85	0.0032	↑,**	5.79±1.11	7.765±1.42	0.0653	↑	
														
**Th1-**	**IL-12p70**	0.28±1.54	1.13±0.12	0.2590	↑	0.00	0.35±4.15	0.3583	↑	n/a	n/a			0.80
	**IL-12p40**	0.00	1.29±1.87	0.0001	↑,***	0.00	10.11±0.96	0.0027	↑,**	79.95±21.42	137.56±101.40	0.1951	↑	0.40
	**IFN-γ**	29.14±2.37	370.51±2.37	0.0001	↑,***	1167.28±537.47	1303.36±239.04	0.3873	↑	n/a	19.34±31.85	0.1761	↑	7.80
	**IL-18**	n/a	29.01	0.1471	↑	0.00	16.42±28.18	0.1370	↑	257.06±63.61	446.86±130.22	0.0429	↑,*	4.90
**Th17-**	**IL-23**	797.37±2302.83	841.56±102.00	0.4904	↑	385.78±240.83	873.61±1501.49	0.3473	↑	2490.27±1052.27	6735.88±7728.16	0.1996	↑	
	**IL-6**	805.28±47.70	9525.56±623.45	0.0013	↑,**	18371.47±1639.82	40165.42±16800.51	0.1047	↑	n/a	67.98±92.86	0.1357	↑	5.50
	**IL-17**	0.68±0.97	4.27±0.99	0.0338	↑,*	1.42±1.53	1.84±1.72	0.4114	↑	n/a	2.056±3.56	0.1870	↑	1.60
**Th2-, B-**	**IL-4**	0.00	25.59±0.33	0.0000	↑,**	47.58±5.25	10.20±7.87	0.0153	↓,*	n/a	n/a			0.80
	**IL-5**	0.00	7.34±0.0275	0.0275	↑,*	2.08±1.39	10.22±7.39	0.1325	↑	n/a	n/a			1.60
	**IL-13**	0.00	11.02±3.09	0.0198	↑,*	5.40±1.73	18.21±4.12	0.0279	↑,*	n/a	n/a			1.60
	**IL-10**	15.52±4.10	281.75±18.50	0.0013	↑,**	501.92±25.65	1155.28±843.26	0.1939	↑	n/a	n/a			1.60
**Treg**	**TGF-β1**	27.64±54.11	426.20±246.13	0.0774	↑	32.067±1114.90	0.00	0.4245	↓	780324.50±172730.67	572900.07±112714.06	0.0782	↓	17.10

Mast cell proteinase-1(MCP-1), MCP-5, macrophage inflammatory protein-1 beta (MIP-1β), regulated upon activation, normal T Cell expressed and secreted (RANTES), matrix metalloproteinase-9 (MMP-9), interleukin-1 beta (IL-1β), IL-4, IL-5, IL-6, IL-10, IL-12p40, IL-12p70, IL-13, IL-17, IL-18, IL-23, interferon-γ (IFN-γ), and transforming growth factor-β1 (TGF-β1) were measured using a multiplex sandwich-ELISA assay (Aushon Biosystem Billerica, MA, USA).

N/A: not able to detect.

LDL: Lowest detectable level.

↑: Production of cytokines (mean) in St^−^ group is ≥50% higher than the St^+^ group.

↓: Production of cytokines (mean) in St^−^ group is ≤50% higher than the St^+^ group.

Unpaired student’s two-tailed t-test was used for statistical analysis.

*P<0.05, **P<0.001, ***P<0.0001.

In the systemic immune system, we observed a striking increase in several chemokines in cultured media of splenocytes isolated from *St14^−^* mice, namely MCP-1, MCP-5, MIP-1β, RANTES and MMP-9. In addition to the increase of proinflammtory T/B cytokines, we also detected a significant decrease in the Th2 polarizing cytokine IL-4 (Table 1). Similarly, the production of TGF-β1 was decreased in splenocyte cultures from *St14^−^* mice compared with cultures from *St14^+^* mice, however, this change was not statistically significant. A similar change in TGF-β1 concentration was also detected in the serum. Th2 cell cytokines such as IL-4, IL-5, IL-10 and IL-13 were below the level of detection in serum (Table 1). However, a consistent and significant increase in proinflammatory cytokines and chemokines was seen in the serum from *St14^−^* mice compared with controls.

Little change in the tested cytokine and chemokine levels were found in either the DLN or spleen of AAV2-Cre mice compared to the control AAV2-LacZ mice (data not shown).

### 5. Decreased salivary flow rate correlates with decreased transepithelial electrical potential in the salivary glands of AAV2-Cre treated mice

**Figure 4:**
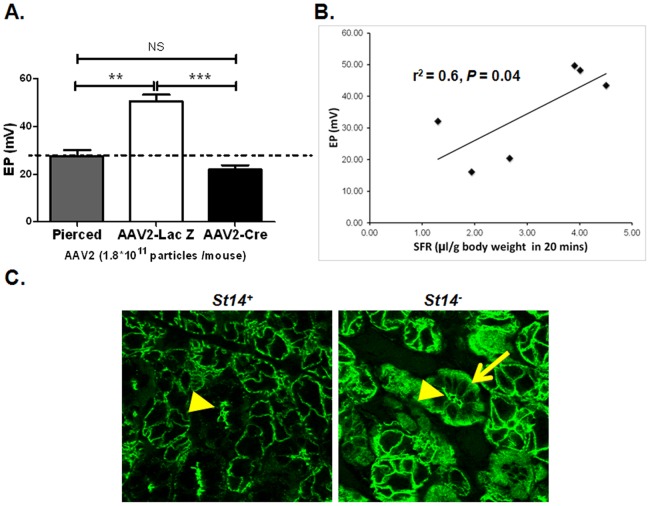
Decreased epithelial electrical potential as well as changes in protein distribution in mice with acute salivary gland-specific ablation of matriptase. Electrical potential (EP) of salivary glands was measured 22 weeks post-AAV2 delivery to glands in live AAV2-Cre (*N = *5) and AAV2-LacZ (*N = *3) mice. Membrane potential of pierced ducts of *St14^LoxP/LoxP^* mice (*N = *2) was used as a control for impaired ductal epithelium. (A) Local depletion of matriptase in SMG from AAV2-Cre animals resulted in a significant EP decrease compared with SMG from AAV2-LacZ control mice. The EP of AAV2-Cre mice was similar to that of pierced glands. (B) Correlation between saliva production and EP of salivary glands from AAV2-Cre mice (*N = *3) and AAV2-LacZ mice (*N = *3). Data shown are the mean ± SEM for each group. Statistical significance was determined using unpaired Student’s *t* test and Pearson’s rank correlation test, respectively. NS, *P = *0.1978, **P = 0.0044, ***P<0.001. (C) Changes in distribution of proteins associated with tight junctions or salivary gland function induced by the loss of matriptase were visualized by immunofluorescent staining of paraffin-embedded SMG tissue samples from *St14^–^* and *St14^+^* control mice 28 to 40 weeks of age (*N = *2 both groups). Representative confocal images are shown. Apical staining of ductal cells for claudin 3 is shown by arrowheads in both *St14^+^* and *St14^−^* animals. An increase in the signal for claudin 3 was detected in the cytoplasm and basal membrane of ductal cells in *St14^–^* mice compared with *St14^+^* mice, as indicated with arrow; original magnification 40X.

Maintaining an impermeable epithelial barrier is critical for the salivary gland function. In order to analyze changes in the epithelial permeability of the salivary glands, we measured the *in vivo* EP in SMG from AAV2-Cre and AAV2-LacZ mice. AAV2-LacZ treated mice had an EP value similar to that of untreated mice (data not shown). In contrast, the EP (mean ± SEM) in the SMG of AAV2-Cre treated mice was significantly reduced compared with AAV2-LacZ mice (21.9±1.9 mV, 50.6±2.7 mV, respectively; *P*<0.001) (Figure 4A). The reduced EP in the AAV2-Cre mice was similar to the EP of mice in which the epithelial monolayer of the duct was disrupted by piercing with a cannula (27.5±2.5 mV) (Figure 4A) suggesting the decrease is related to a loss of barrier function. Furthermore, comparison of SFR data with EP results in each individual mouse indicated a statistically significant correlation between saliva production and EP of the salivary glands (r^2^ = 0.6, P = 0.04). (Figure 4B).

### 6. *St14^−^* mice have altered expression of tight junction protein claudin 3

In addition to the significant immunological changes noted in mice with embryonic deletion of matriptase, the above data also demonstrate that acute local inhibition of matriptase in adult animals is sufficient to alter the EP of the salivary glands and decrease the fluid movement of secretory epithelia both *in vivo* and *ex vivo*. These changes are likely the result of alterations in expression/localization of tight junction proteins and proteins involved in fluid movement in the salivary glands. To further investigate the changes in cell morphology associated with the loss of gland function, immunofluorescent confocal imaging was used to study the distribution of proteins previously associated with tight junction. The most significant change observed in the SMG of *St14^−^* and *St14^+^* mice was the distribution of claudin 3. *St14^−^* mice showed a localization of claudin 3 in the cytoplasm and on the basolateral membrane of ductal cells in *St14^−^* mice compared with an apical localization of this protein in *St14^+^* control mice (Figure 4C). No significant changes were detected in the localization of other cell-cell junction proteins such as ZO-1, (Figure S2). In addition no change was observed in ion channels associated with salivary gland function such as TMEM16A, MaxiK, or NKCC-1 (data not shown). Claudin-2 was not detected in the SMG in either *St14^−^* or *St14^+^* mice (data not shown), which was corresponding to previous report [32], [33].

### 7. Primary sjögren’s syndrome patients display decreased matriptase mRNA in the minor salivary glands

**Figure 5:**
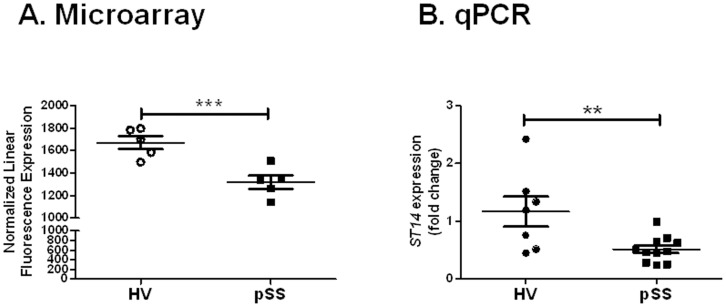
*ST14* expression is decreased in minor salivary glands from primary Sjögren’s syndrome patients. *ST14* gene expression was detected in minor salivary gland biopsies from healthy volunteers and pSS patients by (A) microarray or (B) quantitative PCR (qPCR) analysis. pSS patients (*N = *5 for microarray or *N = *11 for qPCR) displayed a significant decrease of *ST14* gene expression compared with healthy volunteers (*N = *5 for microarray or *N = *7 for qPCR; ***P = 0.003, **P = 0.0093). Data shown are the mean ± SEM for each group. Statistical significance was determined using an unpaired Student’s *t* test.

The above data suggest a connection between compromised epithelial barrier formation and the initiation of a pSS-like disease in mice. To determine the expression of matriptase in pSS patients, total RNA was isolated from the minor salivary glands of pSS patients and gender-matched healthy volunteers and analyzed by both microarray and qPCR. Compared with healthy volunteers, pSS patients had a statistically significant (P = 0.03) decrease in normalized fluorescence intensity signal (mean ± SEM) for matriptase (1671±58 and 1319±61, respectively; *P* = 0.003) (Figure 5A). This observation was further confirmed by testing the matriptase expression level in the minor salivary glands by qPCR. Indeed, the expression of *ST14* (fold change) in the minor salivary glands of pSS patients was decreased by 50% compared with gender-matched healthy volunteers (0.51±0.07 and 1.17±0.26, respectively; *P* = 0.0093) (Figure 5B).

## Discussion

Primary Sjögren’s syndrome is a complex disease characterized by exocrine gland dysfunction, glandular epithelitis, and elevated systemic autoantibodies [1], [34]. However, there is a lack of correlation between lymphocytic infiltrations and dysfunction of exocrine glands [35], implying that additional non-immune factors also may play a role in the symptoms of the disease.

This study suggests that barrier function is important in gland function and that the loss of function may not be a direct effect of inflammation but require changes in the epithelia that result in loss of gland function. The combined data of the embryonic and acute models also suggest the inflammation is likely caused by the loss of matriptase in other non AAV-Cre transduced cell types, possibly outside the salivary gland. Non-salivary gland initiated immune response with associated loss of gland activity are reported in other animal models with primary Sjögren’s syndrome like phenotypes such as the thyroid specific IL-12 transgenic mouse and the Ro60 peptide immunization study (19, 24). Our study followed the mice for 22 weeks post vector delivery with little change in immune activation, many other mouse models of Sjögren’s require the mice to be at least 9 to 13 months of age to develop markers of systemic immune activation [36], [37]. Although it is not clear what is triggering the change in matriptase in patients, the acute depletions of matriptase via AAV-cre suggests that loss of gland function could occur without direct immune activation. Further study will be required to identify the cell type (s) and trigger associated with the induction of immune activation in the embryonic K/O mice and if matriptase activity is effected by immune activation.

Previous studies have reported that matriptase plays a critical role in maintaining homeostasis of multiple epithelia, including the epidermis and intestine and dysregulation of the finely balanced proteolytical activity of matriptase has severe pathophysiological consequences [13], [18], [38], [39]. Indeed, matriptase is reported to play a pivotal role in the formation and integrity of the intestinal epithelial barrier [40]. In the intestine loss of matriptase was associated with enhanced expression and incorporation of the permeability-associated, leaky tight junction protein claudin-2 at intercellular junctions.

Claudin-2 is not reported to be expressed in the salivary gland [32], [33], which we confirmed, however we did detect an alteration in the distribution of claudin 3 in the ductal cells of the matriptase deficient mice. Other TJ protein such as ZO-1 was not changed in *St14^−^* mice. Intact tight junctions are necessary for gland function. In addition to regulating paracellular fluid movement, they also help to maintain the polarization of the plasma membrane components separating the apical and basolateral membrane components [41]. Thus, the change in claudin 3 distribution observed in the *St14^−^* mice is also likely central to the decreased EP and loss in the salivary gland epithelial ductal barrier function observed in the matriptase deficient mice.

How changes in matriptase [42] can affect immune activation is not clear. A likely hypothesis is that changes in epithelial permeability enhances exposure of the microbiota to underlying mucosal immune cells, resulting in general activation of the immune system. This hypothesis is supported by the dramatic reduction of the CD4+ and CD8+ T cells in the local immune system, leaving the remaining T cells to become effector cells. This may further alter the adaptive immune system, induce a loss of the suppressive function of regulatory T cells, such as Treg and Th2 cells, and create an autoimmune inflammation associated with pSS [1], [5], [43].

Primary Sjögren’s syndrome patients have elevated serum levels of proinflammatory cytokines, such as IFN-γ, IL-12, IL-17 and IL-18 that are thought to contribute to the induction of exocrine gland dysfunction in pSS patients [1], [19], [24], [44]. In this study we also observed a significant increase in lymphocyte infiltration in both the salivary and lacrimal glands, accompanied by an overall proinflammatory response in salivary gland DLN. The cytokine profile observed in DLN cell cultures from our matriptase-deficient mice is in agreement with previously published studies in both pSS patients and in animal models of the disease [19], [24], [44], [45] and in the embryonic matriptase knockout mice may contribute to the effect on acinar cells in the SG.

An imbalance of Th1 and Th2 cytokines caused by decreased circulating Th2 cytokines was found to be associated with pSS [43]. Our data indicate an overall immune activation in matriptase-deficient salivary glands associated with elevation in both Th1 and Th2 cytokines and chemokines. In contrast, cytokine and chemokine analysis of the systemic immune system indicated a shift towards a proinflammatory state with increased levels of Th1 and Th17 cytokines and chemokines, but a decrease in several Th2 cytokines and TGF-β1.

The alterations we observe in Treg cells along with Th2 cytokines in the systemic immune systems of matriptase-deficient mice supports this mode of induction of pSS via changes of epithelial barrier function, secretory activity of the cells, and an immune homeostasis.

Interestingly, acute ablation of matriptase from salivary glands of adult mice did not lead to immunological changes at the local or systemic level. This suggests that loss of barrier function in the salivary gland alone may not be sufficient to initiate the immunological changes that characterize pSS, aligning with the general notion of pSS as a systemic autoimmune disorder and epithelitis. In agreement with this, the MMTV-Cre transgene used to knockout matriptase is reported to be active in many tissues including secretory epithelia, skin, or the lung which may be the source for initiation of the immune response [46].

Analysis of matriptase expression in pSS patients showed significantly decreased levels of *ST14* mRNA compared with healthy volunteers. This observation further suggests a possible connection between matriptase expression, epithelial barrier dysfunction, and loss of gland function. It has been reported that matriptase-deficient humans, among other manifestations, suffer from a wide spectrum of symptoms (corneal opacity, photophobia and tooth abnormalities) that all could be linked to compromised epithelial barrier function [47], [48]. However, all examined individuals were children or adolescents, and the direct association between matriptase deficiency and development of pSS, which is typically diagnosed much later in life, has not be reported.

In conclusion, our study presents a new animal model of pSS in which the onset of disease is induced by factors not directly associated with the immune system and suggests an alternative paradigm of autoimmune activation in the disease. Thus, we have shown that a simple defect in matriptase expression and epithelial barrier function suffices to trigger exocrine gland dysfunction and global immune activation.

## Supporting Information

Figure S1 **Matriptase expression in AAV-transduced salivary glands.** (A) In vivo imaging of luciferase expression within the salivary glands following retroductal cannulation. (B) Matriptase expression was quantified using RNA isolated from the SMG following AAV2-LacZ or AAV2-Cre transduction in *St14^LoxP/LoxP^* mice. Relative expression was calculated as ΔΔCT, as described in M&M.
(TIF)

Figure S2 **Detection of ZO-1 expression in matriptase deficiency and control mice.** Immunofluorescent detection of ZO-1 in paraffin-embedded SMG tissue samples from 28 to 40 weeks *St14^–^* and *St14^+^* mice (*N = *2 both groups). Representative confocal images are shown. Apical staining of ductal cells for ZO-1 is shown by arrowheads. No significant difference was detected in *St14^–^* mice compared with *St14^+^* mice. Original magnification is 100X.
(TIF)
