# Supplementary figures and images for: Matriptase Deletion Initiates a Sjögren’s Syndrome-Like Disease in Mice
Source: PLoS One. 2014 Feb 13;9(2):e82852. doi: 10.1371/journal.pone.0082852 (PMC3923742; doi:10.1371/journal.pone.0082852)

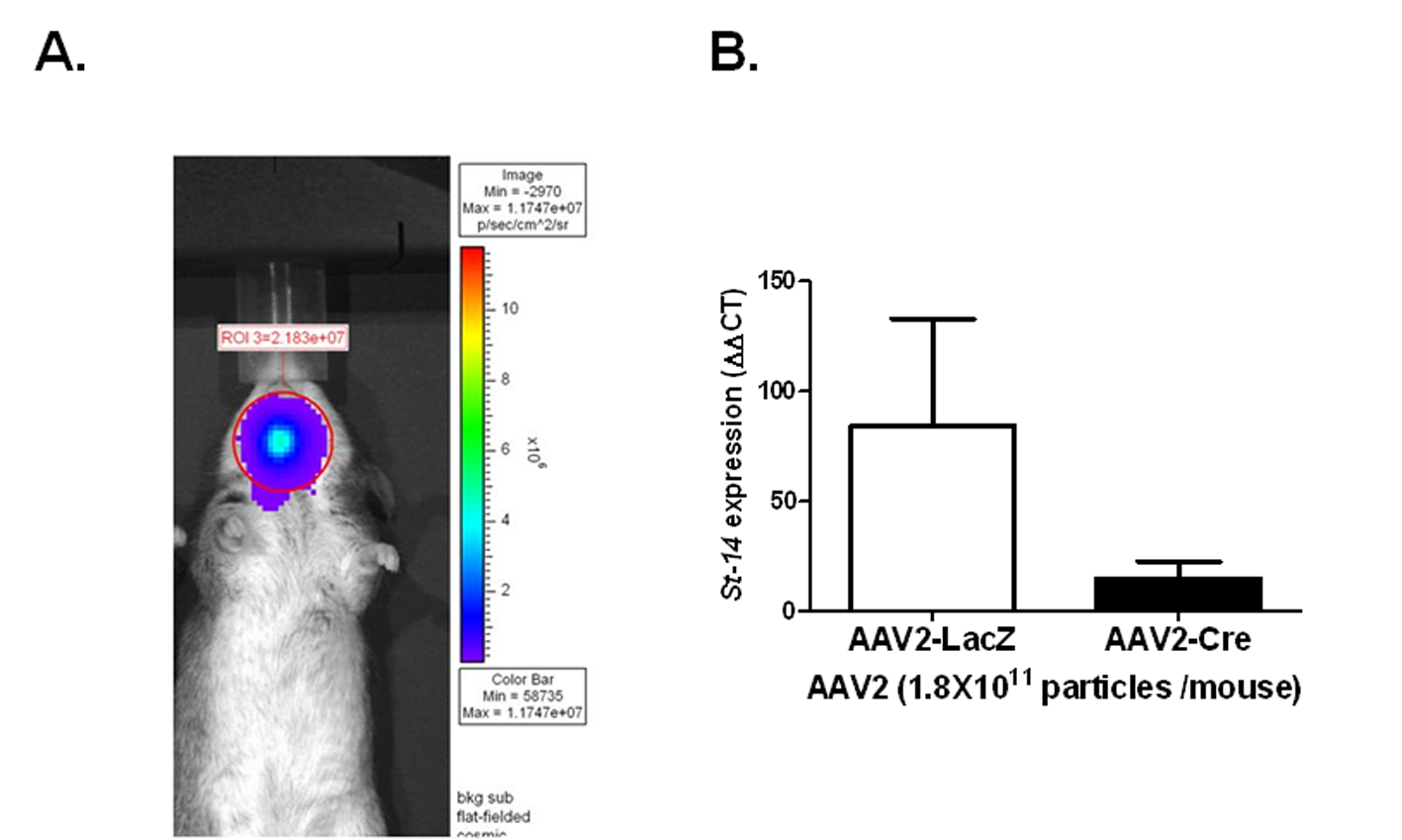

Supplement: Figure S1 — Matriptase expression in AAV-transduced salivary glands. (A) In vivo imaging of luciferase expression within the salivary glands following retroductal cannulation. (B) Matriptase expression was quantified using RNA isolated from the SMG following AAV2-LacZ or AAV2-Cre transduction in St14LoxP/LoxP mice. Relative expression was calculated as ΔΔCT, as described in M&M. (TIF) [file pone.0082852.s001.tif]

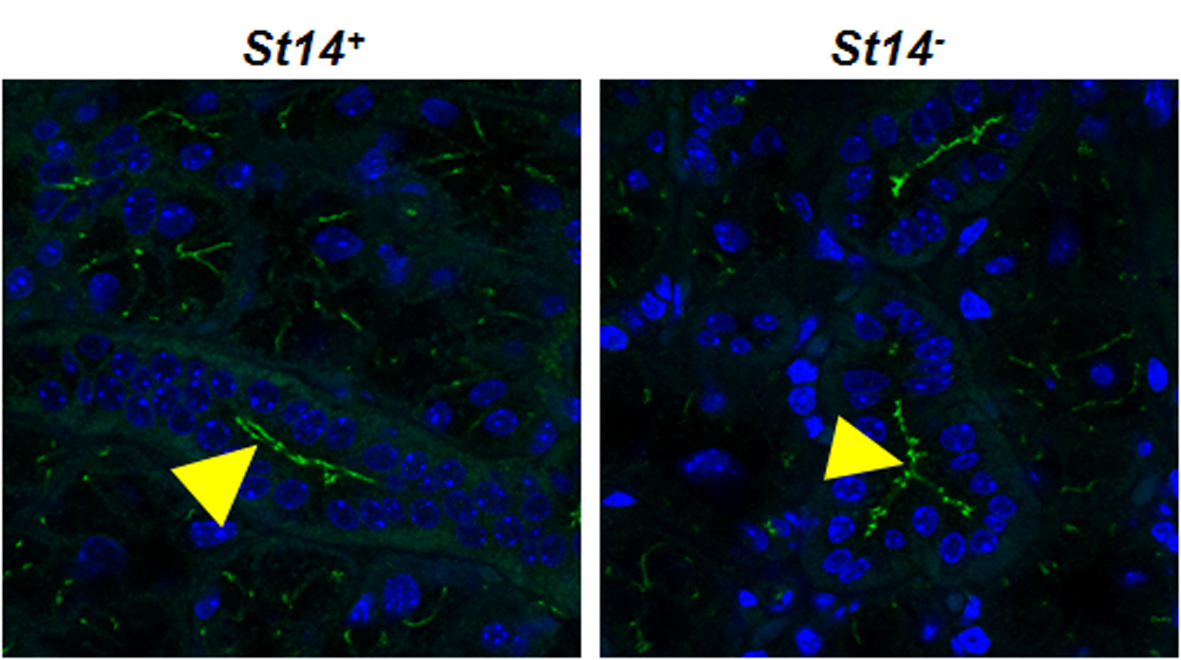

Supplement: Figure S2 — Detection of ZO-1 expression in matriptase deficiency and control mice. Immunofluorescent detection of ZO-1 in paraffin-embedded SMG tissue samples from 28 to 40 weeks St14– and St14+ mice (N = 2 both groups). Representative confocal images are shown. Apical staining of ductal cells for ZO-1 is shown by arrowheads. No significant difference was detected in St14– mice compared with St14+ mice. Original magnification is 100X. (TIF) [file pone.0082852.s002.tif]
